# Developing Community‐Informed Communication Resources to Improve Human Papillomavirus Vaccine Uptake in Tonga

**DOI:** 10.1111/hex.70422

**Published:** 2025-09-07

**Authors:** Yasmin Mohamed, Emma Luey, ‘Ungatea Kata, Isabella Overmars, Ofakiokalani Tukia, Jane Frawley, Afu Tei, Susana Lotulelei, ‘Asinate Toluta'u, Meleane Lomu, Luisa Vodonaivalu, Julie Leask, Holly Seale, Kylie Jenkins, Reynold ‘Ofanoa, Kshitij Joshi, Halitesh Datt, Sonya Sagan, Michelle Dynes, Margie Danchin, Jessica Kaufman

**Affiliations:** ^1^ Murdoch Children's Research Institute Parkville Victoria Australia; ^2^ Department of Paediatrics The University of Melbourne Parkville Victoria Australia; ^3^ Tupou Tertiary Institute Nuku'alofa Tonga; ^4^ Tonga Ministry of Health Nuku'alofa Tonga; ^5^ School of Public Health University of Technology Sydney Ultimo New South Wales Australia; ^6^ School of Public Health The University of Sydney Camperdown New South Wales Australia; ^7^ School of Population Health University of New South Wales Kensington Australia; ^8^ UNICEF Pacific Suva Fiji; ^9^ UNICEF East Asia and Pacific Bangkok Thailand; ^10^ Royal Children's Hospital Parkville Victoria Australia

**Keywords:** communication, health promotion, human papillomavirus vaccine, information needs, Pacific Islands, participatory research

## Abstract

**Introduction:**

Despite high coverage of routine childhood vaccines, uptake of the human papillomavirus (HPV) vaccine in the Pacific Island nation of Tonga has been slow. Culturally appropriate communication resources on the importance, safety, and effectiveness of the HPV vaccine are critical to support acceptance and uptake. To develop these resources, it is important to understand what people want to know. We aimed to explore HPV vaccine information needs among communities in Tonga, to inform a tailored HPV vaccine resource to support uptake.

**Methods:**

We conducted a two phase qualitative study involving stakeholder consultation and feedback to inform the development of a vaccine educational resource. From June to October 2023 (Phase 1), eight focus groups and one interview were conducted with 24 adolescent girls, 32 parents, 15 teachers, seven nurses, and five immunisation program staff in Nuku'alofa, Tonga. Findings informed a flip chart on the HPV vaccine which was qualitatively piloted with nine community members and nine nurses in October 2023 (Phase 2).

**Results:**

Parents, girls, and teachers wanted clear information on the benefits and risks of the HPV vaccine. Immunisation providers lacked culturally specific resources in Tongan, and nurses requested a flip chart. All participants wanted Tongan or Pacific‐specific data on HPV and cervical cancer disease burden. The flip chart was finalised in collaboration with the Tonga Ministry of Health and a local graphic designer. Piloting identified the need for training of nurses on the flip chart, and the potential for the resource to provide information to parents, school students and teachers.

**Conclusion:**

To support HPV vaccine acceptance and uptake in Tonga, we integrated qualitative community and health provider insights to develop and pilot a culturally appropriate resource with locally designed images. Nationwide distribution will now support nurses and community leaders to share information about the HPV vaccine with their communities and additional resources will be developed for adolescent girls.

## 1 Introduction

Improving human papillomavirus (HPV) vaccine acceptance and uptake is a global priority [1]. It aligns with the global strategy to eliminate cervical cancer by 2030 and the Sustainable Development Goals of good health and well‐being (goal 3) and gender equality (goal 5) [1, 2]. Persistent infection with high‐risk HPV types is the primary cause of cervical cancer [3], a disease that causes more than 340,000 preventable deaths annually, most in low‐ and middle‐income countries [4]. Highly effective vaccines against HPV are available, with one dose of any HPV vaccine given in adolescence preventing an estimated 70% of cervical cancer cases [5, 6]. The HPV vaccine is now part of national immunisation programs in 147 countries [7]. However, in 2024 only 31% of eligible girls received one dose, far below the World Health Organization target of 90% [1, 7].

Introduction of the HPV vaccine presents unique challenges and barriers to vaccine acceptance and access [8, 9]. Unlike early childhood vaccines that parents are familiar with, the HPV vaccine is recommended for young adolescents, and it is often only available for girls [7]. The vaccine is delivered in schools and requires parental consent, generally written consent that must be signed at home and returned to the school [10]. HPV itself is also different from most vaccine‐preventable diseases in that it is a sexually transmitted infection [11]. This can lead to cultural resistance and raise parental concerns about fertility and promiscuity in some settings, although there is limited data available from the Pacific region [12]. In addition, because cervical cancer develops later in life, the benefits of the vaccine may not be seen for many years, and cervical screening is still necessary as the vaccine does not prevent all cases of cervical cancer [10, 11, 13, 14].

The Kingdom of Tonga is a Pacific Island Country with around 100,000 inhabitants [15]. The population is predominantly Christian and most people live in rural areas [16]. Cultural communication practices restrict discussions around sexual health, especially in mixed gender settings and between parents and young people [17, 18]. An estimated seven women die each year from cervical cancer in Tonga [19]. At the time of our research, there was no national cervical cancer screening program and only 6% of women had ever been screened [19]. In November 2022, the Ministry of Health introduced the bivalent HPV vaccine free of charge for girls aged 10–17 years [20]; by February 2023 only 20% of eligible girls had received the vaccine [21]. Before HPV vaccine introduction, a quantitative survey of caregivers in Tonga estimated that only 37% had heard of the HPV vaccine [22]. As such, the Ministry of Health launched a communication campaign that included radio and television campaigns, nurse training, and community awareness sessions to accompany HPV vaccine introduction. As limited communication materials were available in Tongan or with comprehensive input from communities, the Ministry requested additional support to increase vaccine uptake. The vaccine was primarily delivered by reproductive health nurses in schools, with parental consent. From early 2023, the country shifted to a one‐dose schedule for 10‐year‐old girls, with a catch‐up campaign for those aged 11–14 years.

As part of a comprehensive multicomponent strategy, tailored and effective communication about the HPV vaccine is essential to drive vaccine acceptance and address misinformation [23, 24]. Communication strategies need to target key stakeholder groups including health workers, community leaders, parents, and adolescent girls themselves [10, 13]. Because the vaccine is delivered in schools, teachers are also relevant stakeholders. To develop culturally appropriate communication materials tailored to the local context, it is important to explore the information needs of the population through formative qualitative research [25]. Qualitative research methods enable in‐depth exploration of community concerns and experiences [26]. While some HPV vaccine information resources are available in Tonga, the Ministry of Health felt that additional resources specific to Tonga would be beneficial to raise awareness and address vaccine concerns and misinformation. There are currently no published studies that explore what information and HPV vaccine resources Tongan communities want. To fill this gap, the Murdoch Children's Research Institute (MCRI) partnered with the Tonga Ministry of Health, UNICEF Pacific, and the Tupou Tertiary Institute (TTI) to explore HPV vaccine information needs among parents, adolescent girls, teachers, nurses, and immunisation staff in Tonga, to inform the development of a tailored and culturally appropriate vaccine information resource.

## 2 Methods

### 2.1 Study Design

**Figure 1:**
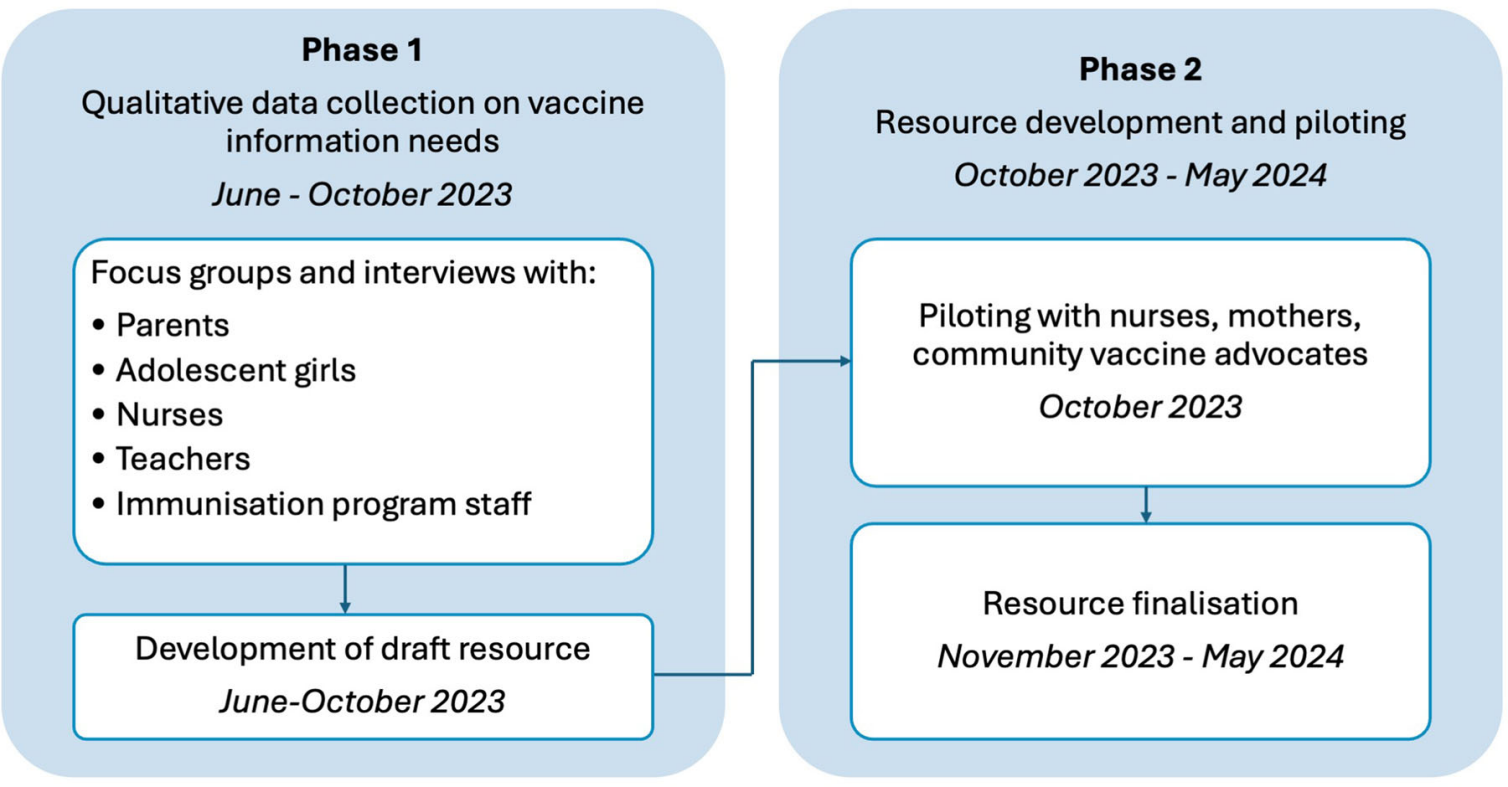
Overview of study phases and activities.

We conducted a two‐phase qualitative study involving stakeholder consultation and feedback to inform the development of a Tongan vaccine educational resource (Figure 1). Phase 1 also explored the social and behavioural drivers of vaccination—these results are presented elsewhere [27]. This study was part of a broader program of work, the Vaccine Champions program, implemented by MCRI and the Ministry of Health from March 2023 to support rollout of the HPV vaccine. The Vaccine Champions program trains community leaders and health workers to address misinformation and become vaccine advocates in their communities [28, 29].

### 2.2 Participant Recruitment

#### 2.2.1 Phase 1

We included stakeholders at all levels of the immunisation program including adolescent girls, parents, teachers, reproductive health nurses, and immunisation program staff from the Ministry of Health. We recruited older adolescent girls aged 15–17 years due to ethical considerations and because this age group was eligible for the HPV vaccine during the initial vaccine rollout.

We recruited parents and adolescent girls using convenience and snowball sampling. The local research team contacted potential participants using telephone calls, Facebook messenger, or face‐to‐face visits, telling them about the study and inviting them to attend a focus group or share the study information with their networks. To recruit teachers, the Ministries of Health and Education and Training sent letters to school principals asking them to invite a teacher to participate. Schools were purposively sampled to ensure participation from both government and church schools, and from the different regions of the main island of Tongatapu. Nurses and immunisation program staff with knowledge and experience of the HPV program were purposively identified and invited to participate by contacts within the Ministry of Health.

#### 2.2.2 Phase 2

The vaccine resource was piloted with mothers, nurses, and community vaccine advocates (Vaccine Champions). Members of the local research team worked with the Ministry of Health to organise a community awareness session run by two nurses at a local community centre. Mothers in the community were invited to attend by local researchers. Immediately after the community awareness session, the two nurses were invited to take part in a group interview and the mothers were invited to participate in a focus group. Community vaccine advocates who were already being interviewed as part of an evaluation of the Vaccine Champions program were asked additional questions about the resource during their group interview. Nurses from the Ministry of Health working in the immunisation program were invited to take part in a focus group.

### 2.3 Data Collection

Two male and five female Tongan data collectors from TTI led the data collection, supported by experienced female Tongan and Australian‐based researchers. Data collectors were chosen based on their connections to their communities and community engagement expertise. The first and second authors led a 1‐day training for all data collectors on the study objectives, qualitative research methods, research ethics, and informed consent procedures. Four of the female data collectors attended additional training on research with adolescents. The local research team indicated that it would be culturally inappropriate to discuss sexual health with males and females together [30]. Therefore, female data collectors facilitated focus groups with female participants and the two male data collectors led discussions with the group of fathers on the HPV vaccine. Female researchers facilitated a mixed group of fathers and mothers on routine vaccines for young children; this group did not discuss the HPV vaccine or any aspects of sexual health. A note taker was present for all discussions.

All focus groups and interviews were opened with a prayer led by the Tongan facilitator or a participant [31], and refreshments and travel reimbursements were provided.

#### 2.3.1 Phase 1

We used focus groups and semi‐structured interviews to explore vaccine information needs, with a focus on the HPV vaccine. The team developed the question guides in English, with a local researcher checking them for cultural appropriateness. The guides were translated into the Tongan language using a collaborative group process involving several members of the local research team.

Focus groups with teachers, parents, and adolescent girls were conducted in Tongan and took place in a private location at TTI. Discussions with nurses and immunisation staff were held in English at the Ministry of Health. A debrief session was held after all focus groups and interviews to discuss key findings, any challenges that arose, and what could be explored in future groups.

#### 2.3.2 Phase 2

During piloting, participants were shown the vaccine information resource either in a community awareness session led by a nurse or informally by the research team. We developed qualitative interview guides based on the Patient Education Materials Assessment Tool for Printable Materials (PEMAT‐P), with a focus on the understandability and actionability of the resource [32]. To ensure that the final resource was relevant and useful for the Tongan context, we added questions to explore its cultural appropriateness [33].

The mothers' focus group and the interview with the two nurses running the community awareness session were held at a local community centre. The focus group was conducted in Tongan and the nurse interview in English. The focus group with community vaccine advocates took place in a private location at TTI, and the focus group with nurses was held at the Ministry of Health.

#### 2.3.3 Data Management

All focus groups and interviews in phase 1 and 2 were audio recorded with consent, with audio files deleted from voice recorders once transferred onto secure MCRI servers. A Tongan company was contracted to transcribe the data verbatim and translate it into English. Focus group facilitators checked the translated transcripts for accuracy. Audio files and transcripts were stored on secure servers at MCRI, separately from consent forms.

#### 2.3.4 Ethical Approvals

We obtained ethical approval from the Tonga National Health Ethics and Research Committee (MH 53:02) and the Royal Children's Hospital Melbourne Human Research Ethics Committee in Australia ((#84863). All participants provided written informed consent, and parental consent was additionally obtained for all adolescents.

### 2.4 Data Analysis and Resource Development

#### 2.4.1 Phase 1

The first author analysed the data using inductive content analysis [34, 35] with NVivo software (QSR International Pty Ltd), developing initial codes and themes using an iterative process. Three senior members of the research team, including an experienced Tongan researcher reviewed the codes and themes before they were finalised to ensure reliability and cultural relevance. As similar ideas began to emerge repeatedly across interviews and focus groups, we believed that data saturation had been reached.

Based on the insights from the focus groups in phase 1, we developed the vaccine information resource: a flip chart about the HPV vaccine and routine childhood vaccines. The MCRI team developed a mock‐up of the flip chart for informal review by Vaccine Champions and Ministry of Health nurses in August 2023. The team made updates based on the feedback and produced a draft flip chart in English using graphics that were freely available on the World Health Organization website. Staff from TTI translated the text of the flip chart into Tongan, and nurses from the Ministry of Health reviewed the translation.

#### 2.4.2 Phase 2

Pilot data were analysed by the first author using inductive content analysis with NVivo software (QSR International Pty Ltd.). The team from MCRI updated the flip chart content based on the findings from the pilot. The layout and design were redeveloped by an Australian graphic designer, and a Tongan graphic designer drafted images of Tongan characters to be included. TTI made the required updates to the Tongan text and staff from the Ministry of Health reviewed the translation.

## 3 Phase 1 Results

### 3.1 Study Participants

The focus groups and interviews with adolescent girls, parents, teachers, nurses, and immunisation program staff in phase 1 are outlined in Table 1. Most participants were female (74/83; 89%) and nine were male (11%).

**Table 1:** Participants included in Phase 1.

Participant group	Focus groups and interviews (number of participants in each)	Total number of participants (gender)
Reproductive health nurses	1 (7)	7 (F)
Immunisation program staff	2 (1, 4)	5 (F)
Mothers of girls aged 10–17 years	2 (17)	17 (F)
Fathers of girls aged 10–17 years	1 (6)	6 (M)
Parents of children aged < 5 years	1 (9)	9 (6 M, 3 F)
Primary school teachers	1 (7)	7 (F)
Secondary school teachers	1 (8)	8 (F)
Adolescent girls aged 15–17 years	4 (6)	24 (F)
Total focus groups/interviews (participants)	13	83 (74 F, 9 M)

### 3.2 Findings

We categorised the data into two main themes: (1) what participants wanted to know and (2) how participants wanted to learn about the HPV vaccine. These are explored in more detail below, followed by a description of how we developed the resource from the findings.

#### 3.2.1 What Participants Wanted to Know

Adolescent girls, mothers, and teachers described how they had recently learned of the HPV vaccine through radio campaigns, work colleagues and nurses at school. Adolescent girls specifically asked for information about the benefits of the HPV vaccine in protecting against cancer and how the virus is spread.

There is a need to explain to the youth about protection against cancer and infections in the body.

(Adolescent; FG2; Phase 1)

Parents, girls, and teachers wanted information on vaccine benefits, side effects especially long‐term side effects, effectiveness, and length of protection.

I just want to know whether the vaccine is worth it, I want to know if there has been anyone that has been cured from the virus and if anyone has had any benefit from the vaccine.

(Adolescent girl; FG2; Phase 1)

While female participants from all groups had some knowledge of the existence of the HPV vaccine, many of the fathers had never heard of it before the focus group. Most fathers wanted more information about the HPV vaccine, including how long it would protect their daughter for. Some fathers wanted this information so that they could support their daughters to make informed decisions about vaccination.

Parents wanted to know the specific ingredients of the vaccine and how it had been previously tested. Both mothers and fathers asked for more information about why the vaccine was introduced in Tonga and its use elsewhere.

A background story about this vaccine will be helpful, talking about how this vaccine came to Tonga, where it came from, places that has already used it and more information. This would be very helpful for the people when they do their research and know that this vaccine is safe knowing that it is [used] worldwide.

(Mother; FG2; Phase 1)

A small number of parents and teachers questioned why the HPV vaccine was given to young girls who were not married and therefore not sexually active. In addition, the link between vaccinating in early adolescence to protect against cervical cancer later in life was not always well understood.

Why is it that you guys only give [HPV] vaccines to this age [10–14 years] while from all the reports I hear the people that deals with this virus the most are people that are married, have kids and are much older?

(Mother; FG1; Phase 1)

Nurses and immunisation staff felt the community wanted to know that there was “no cost to fertility” (Immunisation staff; FG2; phase 1). Some wanted images clearly showing the location of the cervix, although the sensitivity of this image in groups where men were present was acknowledged. Participants also wanted data and statistics specific to Tonga.

I think it would help a lot if we had the facts or statistics on the number of deaths caused by HPV [in Tonga].

(Nurse; Phase 1)

#### 3.2.2 How Participants Wanted to Learn About the HPV Vaccine

Participants had many suggestions for providing information to the community about the HPV vaccine including radio shows, TV programs, videos on YouTube, and community outreach. Parents, adolescent girls, and teachers overwhelmingly wanted information that was delivered face‐to‐face by trained health workers and staff from the Ministry of Health.

It would be most effective to have the Ministry of Health do outreach programs to explain the importance of HPV.

(Adolescent girl; FG2; Phase 1)

Comprehensive resources in Tongan were seen as useful, however they needed to be supported by in‐person discussions with nurses or doctors. Some parents talked about the need for resources specifically for adolescent girls, and others highlighted the importance of personal stories from community members who had received or given their daughter the HPV vaccine.

They should have a representative from the community as proof that this vaccine is safe and helpful.

(Mother; FG2; Phase 1)

Nurses and immunisation program staff felt that they had sufficient information about the HPV vaccine but that resources in Tongan would be helpful for effectively conveying this knowledge to the community.

The information is enough, it's the way it is presented to the community—it's a bit difficult trying to translate from English.

(Nurse; Phase 1)

Translating information about the HPV vaccine from English into Tongan was identified as a challenge, with nurses explaining that there was no direct translation of the word “cervix” into Tongan. Nurses felt that a flip chart would be helpful to support their community awareness sessions. Nurses and immunisation staff also felt that more training on vaccine communication and counselling parents about vaccination would be helpful.

Adolescent girls were shown four options for resources about the HPV vaccine: a simple one‐page brochure, a detailed folded brochure, a video, and a comic. There was a mix of preferences for the brochures and the video, with the largest number of girls (11/24) favouring the animated video.

#### 3.2.3 Resource Development

**Figure 2:**
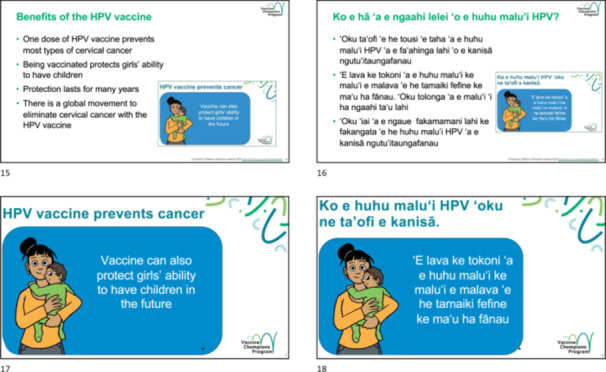
Example of community‐facing (bottom) and presenter‐facing (top) images from the draft flip chart used for piloting in English (left) and Tongan (right).

While study participants suggested a few different options for potential resources, the study team and Ministry of Health felt that a flip chart should be prioritised to support the nurses in their awareness sessions. Nurses requested a flip chart on the HPV vaccine and community members wanted face‐to‐face conversations about the vaccine led by a health worker. A video on the HPV vaccine for adolescent girls is planned as a future activity. The flip chart provided information about vaccines in general and answered common questions and concerns about the HPV vaccine. It was designed to be used by community members such as Vaccine Champions as well as by nurses involved in the immunisation program. The flip chart had a page with a single image and succinct key messages that faced the audience, and a corresponding page with information in dot points for the person running the session (Figure 2). To ensure cultural appropriateness, the diagram of the cervix was added at the back of the flip chart so that it could be avoided if it was not appropriate for a particular audience.

Several key information needs identified in the phase 1 qualitative research were incorporated into the flip chart. These included:

1. The long‐term side effects of the HPV vaccine and length of protection.
2. The HPV vaccine is new to Tonga but not new globally.
3. Focus on cancer prevention and preserving fertility.
4. How fully the vaccine protect girls.

## 4 Phase 2 Results

### 4.1 Study Participants

An overview of the participants included in Phase 2 is provided in Table 2. Focus groups and interviews were conducted with a total of 20 nurses, mothers, and vaccine advocates.

**Table 2:** Participants included in Phase 2.

Participant group	Focus groups and interviews (number of participants)	Total participants (gender)
Reproductive health nurses	2 (2, 7)	9 (F)
Mothers of girls aged 10–17 years	1 (9)	9 (F)
Vaccine Champions	1 (2)	2 (F)
Total focus groups/interviews	4	20 (F)

### 4.2 Findings

The overall feedback on the draft flip chart was positive. An overview of suggestions from the nurses and community members is provided in Table 3. Community members and nurses felt that the resource was useful, especially for presenting to small groups of people. Nurses thought that the flip chart could be used to provide information to small groups of parents, school students and teachers:

I think the flip chart is the best way for small groups.

(Nurse; FG1; Phase 2)

**Table 3:** Summary of suggested inclusions and changes to the vaccine information resource from piloting (phase 2).

Participant group	Suggested inclusion/change
Nurses	Colour scheme to match Ministry of Health reproductive health materials Updates to the vaccine schedule to reflect recent changes Clearly stating that the vaccine does not affect fertility
	“Like when we talk about HPV, the first thing people bring up is infertility. These things are what is being brought up by the community” (Nurse; FG2; Phase 2)
Community members (mothers and community vaccine advocates)	More details on the benefits and long‐term side effects of the HPV vaccine
	“A little background on the benefits of [the HPV vaccine] will do.” (Mother; Phase 2) “Some of the parents first ask about the risks and statistics, the side effects and whether the vaccine has been accepted globally. They first needed proof and evidence before allowing their children to be vaccinated.” (Vaccine advocate; Phase 2)
All groups	Inclusion of Tongan or Pacific data on cervical cancer and HPV
	“We need proof of studies that have been done [about the HPV vaccine] so that we can show it to parents. Any statistics and the side effects if any…” (Vaccine advocate; Phase 2)

Participants believed that all the important information about the HPV vaccine and routine childhood vaccines was covered and that the messages were presented clearly. Nurses thought that the flip chart addressed all the concerns and questions they heard in the community.

Yes, the main concerns from the community is the infertility which you already covered and information on [length of] HPV protection.

(Nurse; FG2; Phase 2)

Nurses wanted training on the flip chart ranging from a 1‐day workshop to a simple video demonstrating how to use it. The nurses who presented the flip chart to mothers in the community particularly liked the picture of the cervix as it was helpful to show the audience exactly where the cervix is in the body.

Information collected during the piloting phase led to updates to the vaccine information resource (Table 3). The final Tongan version of the flip chart (Figure 3) has been printed and provided to the Ministry of Health for national distribution. It will be used to support community awareness sessions run by reproductive health nurses and also by community vaccine advocates through continuation of the Vaccine Champions in 2025. Additional examples of community and presenter‐facing pages from the English and Tongan flip charts are included in a Supporting Information.

Figure 3Example of community‐facing (b, d) and presenter‐facing (a, c) images from the final flip chart in English (a, b) and Tongan (c, d).

**Figure:**
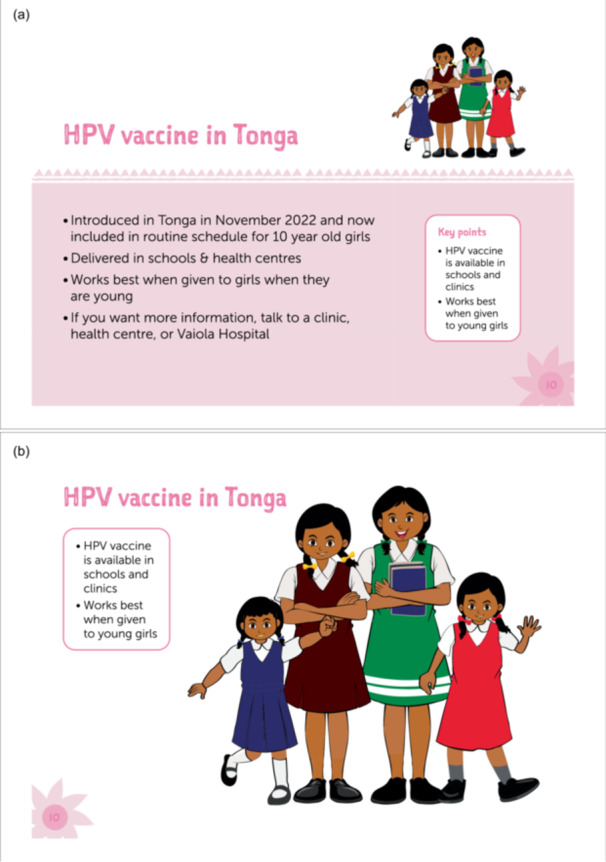

**Figure:**
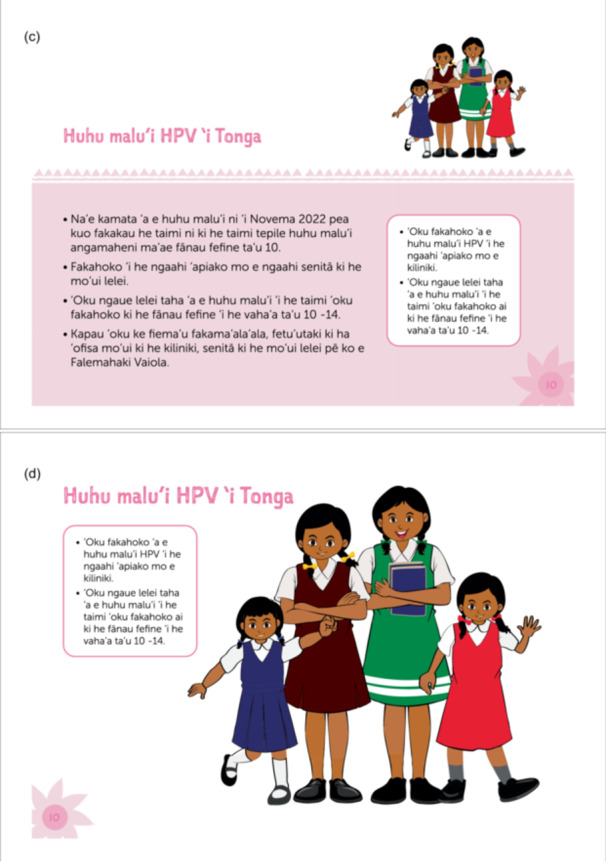

## 5 Discussion

The HPV vaccine information needs among parents, teachers and adolescent girls in Tonga highlight the importance of raising awareness about the vaccine, especially with fathers, and of addressing specific questions and concerns. There was a strong preference for in‐person discussions with nurses or doctors supported by locally tailored resources for both parents and adolescent girls, and participants raised the importance of personal stories from community members who had received the vaccine or vaccinated their girls. Nurses and immunisation program staff wanted culturally tailored resources in Tongan, specifically requesting a flip chart. These insights were used to develop a flip chart that was locally designed, used context‐appropriate images and addressed the key concerns we heard in the community. We integrated feedback from mothers, nurses, and community vaccine advocates into the final flip chart that will be used to share culturally appropriate information about the HPV vaccine in Tonga. Young girls also requested resources for them, favouring an animated video.

Adolescent girls, parents, and teachers wanted specific information about the HPV vaccine including the benefits, long‐term side effects, length of protection, data on vaccine safety and effectiveness, information on how the virus is spread, the disease burden in Tonga, and the rationale for vaccinating young girls who were not sexually active. Studies from a diverse range of settings also demonstrate the need for more in‐depth information about the HPV vaccine among parents and young people [36, 37, 38, 39, 40, 41], and research from multiple countries including Malawi, Papua New Guinea and Trinidad similarly identified concerns about the age of vaccination [37, 40, 41]. Adolescents, parents, and teachers play a key role in vaccine decision‐making and have the right to accurate and appropriate information about the HPV vaccine that addresses their key concerns [27]. Effective communication and information sharing can promote HPV vaccine uptake [42], especially if it targets relevant groups including teachers, parents, girls and community leaders to enhance community ownership [24].

Similar to a recent systematic review [43], the nurses and immunisation program staff that we spoke to had sufficient information about the HPV vaccine but identified vaccine communication as a challenge and requested additional resources to support their conversations with communities. Health provider recommendations are critical to improving HPV vaccine uptake [44]. In Fiji for example, health workers are seen as trusted sources of information about the HPV vaccine, and play a key role in facilitating decision‐making [45]. Nurses and other health professionals need the knowledge, communication skills and resources to effectively communicate with communities about the HPV vaccine. The Tongan flip chart will support nurses in their community conversations, however further communication training was requested by nurses and should be considered. Insufficient training on how to communicate about the HPV specifically, including with adolescents, is a barrier to HPV vaccination in low‐ and middle‐income settings [8]. Training health workers and community leaders in Fiji on vaccine‐specific communication skills has been shown to effectively increase knowledge and confidence to communicate about vaccines [29].

The flip chart developed during this study framed the HPV vaccine as a way to prevent cancer and preserve fertility. This messaging was perceived to be culturally acceptable to the study participants and is consistent with the existing literature on promoting the HPV vaccine in low‐ and middle‐income countries [8, 42, 46]. A global systematic review of parental acceptance, attitudes and knowledge of the HPV vaccine found that wanting to protect their children against cancer was a significant predictor of vaccine acceptance [47]. Crafting appropriate and impactful messaging around the HPV vaccine for specific population groups can be challenging. A qualitative study in Papua New Guinea found that young people wanted the vaccine described as one to prevent a sexually transmitted infection, whereas older people preferred an emphasis on cancer prevention [36]. This highlights the nuances of communicating about the HPV vaccine and the need to develop messaging with communities to ensure that they are acceptable and appropriate for different audiences and age groups.

A recent review from sub‐Saharan Africa found that the most effective communication strategies to enhance HPV vaccine uptake were those that educated a range of stakeholder groups including adolescent girls themselves [24]. Our flip chart was well received in the piloting and will be used by nurses and community vaccine advocates to share information about the HPV vaccine with parents, teachers, and adolescent girls. However, there remains a need for adolescent‐specific resources on the HPV vaccine in Tonga. A study in Malawi found that adolescent girls who read a magazine specifically designed to share information about the HPV vaccine had greater vaccine knowledge and awareness, and that reading the magazine correlated with increased HPV vaccine uptake [48]. Our study suggests that a video may be the most appropriate resource for young adolescent girls in Tonga; the codesign and development of this video will commence later in 2025.

The flip chart was well received by nurses and community members. The pilot identified a need to train nurses on how to use the flip chart and that the resource could be used to share information with parents, school students and teachers. Our Vaccine Champions program in Fiji trained health workers and community leaders with the knowledge and vaccination communication skills to effectively share information about COVID‐19 and routine childhood vaccines [29]. This program was tailored to support HPV vaccine uptake in Tonga and will continue in 2025, with the flip chart being used to support community conversations about the HPV vaccine. Crucially, the updated program will use a train‐the‐trainer model, where local experts will be provided with the knowledge and skills to train Vaccine Champions themselves. This will ensure the sustainability of the program in Tonga and will build the capacity of local staff in vaccine communication.

While communication interventions such as the development of a locally developed vaccine information resource are an essential part of the HPV vaccine rollout, information and education alone are not sufficient to drive vaccine uptake. Interventions with multiple components and those that target multiple levels are likely to be the most effective at improving HPV vaccine uptake [49]. A broader HPV vaccine campaign is underway in Tonga, with nurse training, media campaigns, face‐to‐face community awareness sessions, and house‐to‐house visits by nurses to address access barriers. Additional strategies to consider include strengthened engagement and education of school principals and teachers, involvement of trusted community leaders, communication training for nurses, and resource development and education for young adolescent girls [9, 11, 23, 24, 42, 44, 49, 50].

### 5.1 Strength and Limitations

One key strength of this study was having a data collection team made up of local researchers with strong community connections, bringing a crucial cultural perspective to the work. Conversely, recruiting study participants through the networks of our data collection team limits the diversity in our included sample and may have included more people with positive views of vaccination. Recruiting through local networks may also have introduced social desirability bias, where included participants may not have felt comfortable sharing negative opinions as freely. Our data collection team was conscious of this and made every effort to ensure that participants felt comfortable to speak honestly. Our study was conducted on the main island of Tongatapu and did not include people from the outer islands where information needs may be different. Tongatapu is a small island with interconnected communities where having a research team that is completely unknown to all participants is unlikely. Having more focus groups with fathers would be beneficial for future research.

## 6 Conclusion

To improve HPV vaccine acceptance and uptake in Tonga, we developed a community‐informed vaccine information resource in the Tongan language with locally designed images and culturally relevant information. We sought the views of adolescent girls, mothers, fathers, teachers, nurses, and immunisation program staff to design a resource that addresses specific community concerns and information needs. The resource will be used by nurses and diverse community vaccine advocates to support in‐person discussions with parents, adolescent girls and teachers to increase awareness and uptake of the HPV vaccine and ultimately contribute to the elimination of cervical cancer in Tonga. Culturally appropriate communication resources should be an integral component of HPV vaccine campaigns and adolescent‐friendly formats should be prioritised. Future work will evaluate the effectiveness of the flip chart in improving knowledge, awareness and attitudes towards the HPV vaccine in Tonga, adapt it to other settings in the Pacific region, and codesign adolescent‐specific resources.

## Author Contributions

**Yasmin Mohamed:** conceptualisation, methodology, investigation, writing – original draft, formal analysis, resources, project administration, visualisation, data curation, software. **Emma Luey:** conceptualisation, methodology, investigation, writing – review and editing, formal analysis, project administration, data curation, validation. **‘Ungatea Kata:** conceptualization, investigation, methodology, validation, writing – review and editing, resources, data curation. **Isabella Overmars:** conceptualisation, investigation, methodology, project administration, writing – review and editing. **Ofakiokalani Tukia:** conceptualisation, methodology, writing – review and editing. **Jane Frawley:** writing – review and editing, conceptualisation, methodology. **Afu Tei:** conceptualisation, methodology, writing – review and editing. **Susana Lotulelei:** investigation, methodology, project administration, writing – review and editing. **‘Asinate Toluta'u:** investigation, methodology, project administration, writing – review and editing. **Meleane Lomu:** investigation, methodology, project administration, writing – review and editing. **Luisa Vodonaivalu:** conceptualisation, methodology, investigation, project administration, writing – review and editing. **Julie Leask:** conceptualisation, methodology, writing – review and editing, funding acquisition. **Holly Seale:** conceptualisation, methodology, writing – review and editing, funding acquisition. **Kylie Jenkins:** conceptualisation, methodology, writing – review and editing, funding acquisition. **Reynold ‘Ofanoa:** methodology, writing – review and editing. **Kshitij Joshi:** conceptualisation, methodology, writing – review and editing. **Halitesh Datt:** conceptualisation, methodology, writing – review and editing, project administration. **Sonya Sagan:** conceptualisation, methodology, writing – review and editing. **Michelle Dynes:** conceptualisation, methodology, writing – review and editing. **Margie Danchin:** conceptualisation, investigation, funding acquisition, methodology, writing – review and editing, formal analysis, supervision, validation. **Jessica Kaufman:** conceptualisation, investigation, funding acquisition, methodology, writing – review and editing, formal analysis, supervision, validation, visualisation, software.

## Ethics Statement

We obtained ethical approval from the Tonga National Health Ethics and Research Committee (MH 53:02) and the Royal Children's Hospital Melbourne Human Research Ethics Committee in Australia (#84863).

## Consent

All participants provided written informed consent, and parental consent was additionally obtained for all adolescents.

## Conflicts of Interest

O.T., A.T. and R.O. are employed bythe Tonga Ministry of Health and involved in the national HPV vaccine program. H.S. has received funding from vaccinemanufactures for investigator driven research and hasconsulted on vaccination for Pfi zer and Moderna. This fundingwas not related to this work. The other authors declare no conflicts of interest.

## Patient or Public Contribution

The design and conduct of this study was informed by the local data collection team, who are part of the communities included in the research (teachers and parents). Our vaccine information resource was informed by our participatory qualitative research with communities, and the piloting allowed us to gain additional insights from parents, community vaccine advocates, and nurses.

## Supporting information

Supplementary file.

## Data Availability

The data that support the findings of this study are available upon reasonable request from the corresponding author. The data are not publicly available due to privacy or ethical restrictions.
