# Supplementary material for: Developing Community‐Informed Communication Resources to Improve Human Papillomavirus Vaccine Uptake in Tonga
Source: Health Expect. 2025 Sep 7;28(5):e70422. doi: 10.1111/hex.70422 (PMC12414434; doi:10.1111/hex.70422)

Benefits of the HPV vaccine

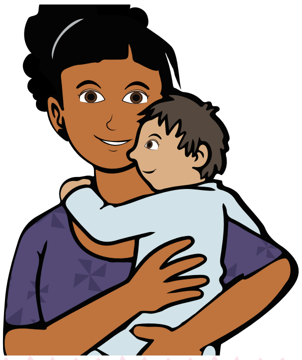

- One dose of HPV vaccine prevents most types of cervical cancer
- Almost all cervical cancer is caused by HPV, and the vaccine decreases HPV by 90%[3]
- Being vaccinated protects girls' ability to have children in the future
- Protection lasts for many years
- There is a global movement to eliminate cervical cancer with the HPV vaccine

Key points

- HPV vaccine prevents cancer
- The HPV vaccine can protect girls' ability to have children in the future

[3] https://www.ncbi.nlm.nih.gov/pmc/articles/PMC4967609/

Benefits of the HPV vaccine

- HPV vaccine prevents cancer
- The HPV vaccine can protect girls' ability to have children in the future

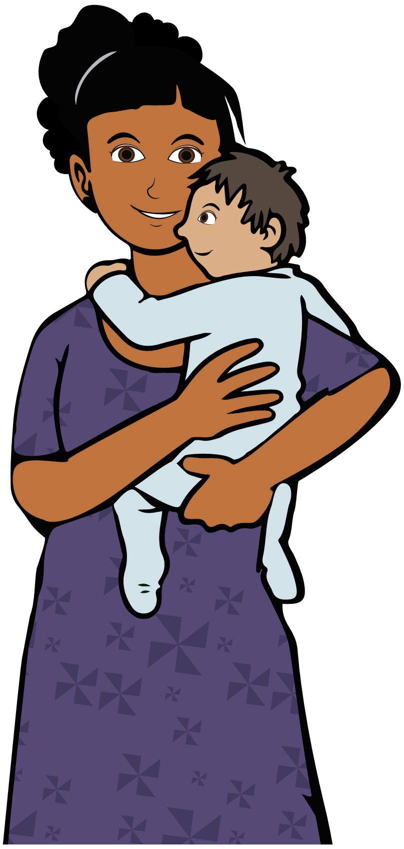

Ko e hā ʻa e ngaahi lelei ʻo e huhu maluʻi HPV?

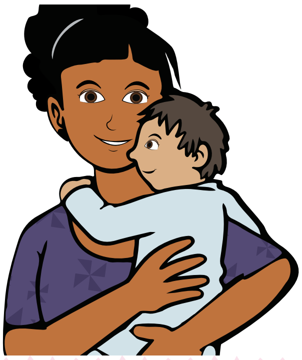

- Ko e foʻi huhu maluʻi HPV ʻe taha te ne lava ʻo maluʻi ha taha mei he faʻahinga ʻe niʻihi ʻo e kanisā ngutu ʻo e taungafanau.
- Ko e kanisā ngutu ʻo e taungafanau ʻoku fakatupu ia ʻe he HPV, pea ko e huhu maluʻi ʻoku ne malava ke maluʻi ʻo aʻu ki he 90% ʻa kinautolu ʻoku kau ki he huhu maluʻi.
- ʻOku ʻikai uesia ʻe he huhu maluʻi ni ʻa e maʻu fānau ʻa e kakai fefine.
- ʻOku tolonga ʻa e maluʻi ʻi ha ngaahi taʻu lahi
- ʻOku ʻi ai ʻa e palani fakamamani lahi ke ʻoua toe ʻasi ʻa e kanisā ni ʻi mamani makatuʻunga ʻi he kau mai e taha kotoa ki he huhu maluʻi HPV.

[3]https://www.ncbi.nlm.nih.gov/pmc/articles/PMC4967609/

- Ko e huhu maluʻi HPV ʻoku ne taʻofi e kanisā.
- ʻOku ʻikai uesia ʻe he huhu maluʻi ni ʻa e maʻu fānau ʻa e kakai fefine.

Ko e hā ʻa e ngaahi lelei ʻo e huhu maluʻi HPV?

- Ko e huhu maluʻi HPV ʻoku ne taʻofi e kanisā.
- ʻOku ʻikai uesia ʻe he huhu maluʻi ni ʻa e maʻu fānau ʻa e kakai fefine.

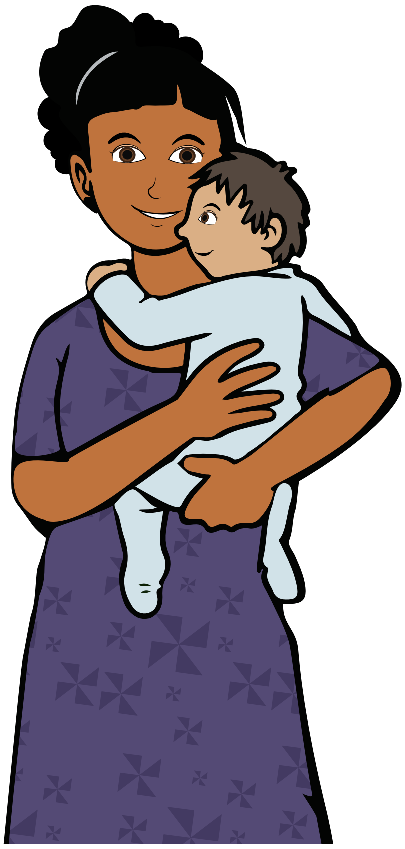

What are the side effects of the HPV vaccine?

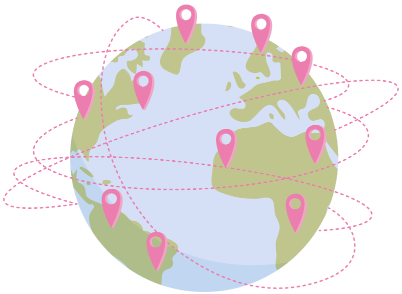

- Common side effects are usually mild:
  - Sore arm
  - Headache
  - Fainting
  - Nausea
  - Fever
  - Dizziness
- Over 500 million doses of HPV vaccine delivered worldwide since 2006
- No evidence of serious or long-term side effects from studies all around the world

Key points

- Common side effects are usually mild:
  - Sore arm
  - Headache
  - Fainting
  - Nausea
  - Fever
  - Dizziness
- Delivered around the world since 2006

What are the side effects of the HPV vaccine?

- Common side effects are usually mild:

  - Sore arm
  - Headache
  - Fainting
  - Nausea
  - Fever
  - Dizziness

- **Delivered around the world since 2006**

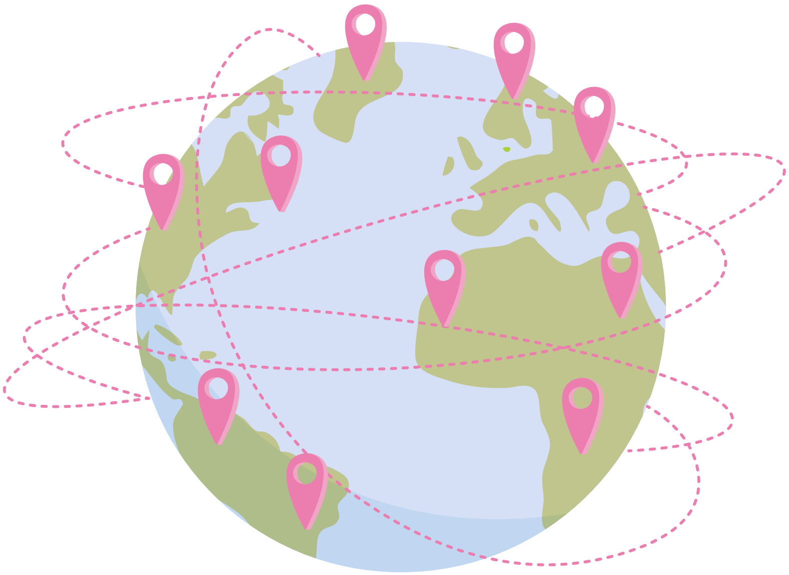

Ko e ha e ngaahi uesia 'o e huhu malu'i HPV?

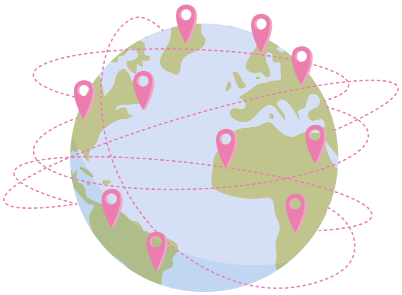

- 'Oku fa'a vaivai pē 'a e ngaahi uesia makehe angamaheni
  - Mamahi 'o e uma
  - Langa 'ulu
  - Pōngia
  - Tokakovi'ia
  - Mofi
  - ninimo
- Na'e laka hake 'i he ngaahi tousi huhu malu'i ki he kanisā ngutu 'o e taungafanau 'e 500 miliona na'e tufaki fakamamanilahi talu mei he 2006
- 'Oku 'ikai ha fakamo'oni ia ki ha ngaahi uesia makehe fakatu'utāmaki pē taimi loloa mei he ngaahi ako 'i he funga 'o e mamani

- 'Oku fa'a vaivai pē 'a e ngaahi uesia makehe angamaheni
  - Mamahi 'o e uma
  - Langa 'ulu
  - Pōngia
  - Tokakovi'ia
  - Mofi
  - Ninimo
- 'Oku fo'ou 'a e huhu malu'i HPV ki Tonga ka 'oku 'ikai fo'ou fakamamani lahi ia.
- Na'e tufaki 'i mamani talu mei he 2006

Ko e ha e ngaahi uesia 'o e huhu malu'i HPV?

- 'Oku fa'a vaivai pē 'a e ngaahi uesia makehe angamaheni
  - Mamahi 'o e uma
  - Langa 'ulu
  - Pōngia
  - Tokakovi'ia
  - Mofi
  - ninimo
- 'Oku fo'ou 'a e huhu malu'i HPV ki Tonga ka 'oku 'ikai fo'ou fakamamani lahi ia.
- Na'e tufaki 'i mamani talu mei he 2006

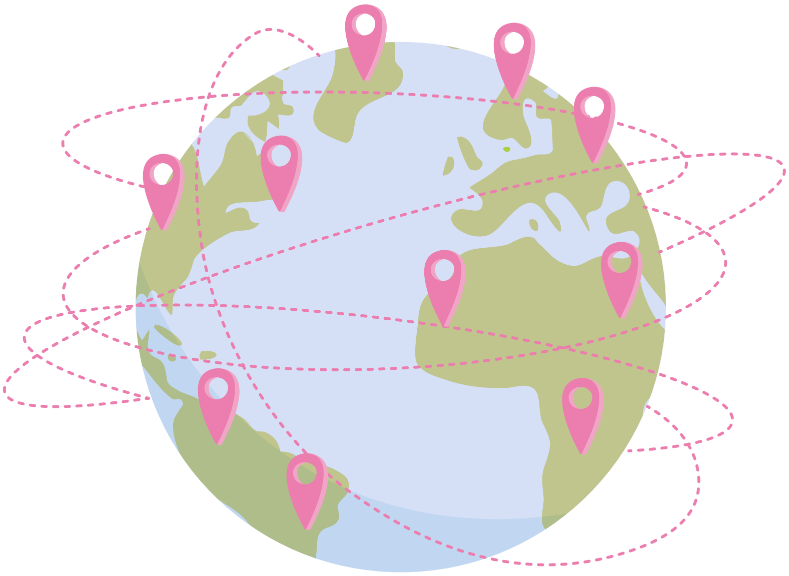

Supplement: Supplementary file 1 — Supplementary file. [file HEX-28-e70422-s001.pdf]
